# Supplementary material for: The Impact of Milk Fat Globule Membrane with Exercise on Age-Related Degeneration of Neuromuscular Junctions
Source: Nutrients. 2021 Jul 5;13(7):2310. doi: 10.3390/nu13072310 (PMC8308682; doi:10.3390/nu13072310)
Supplement: Supplementary file 1 [file nutrients-13-02310-s001.zip › nutrients-1248120-supplementary.pdf]

nutrients

Suuplementary Materials

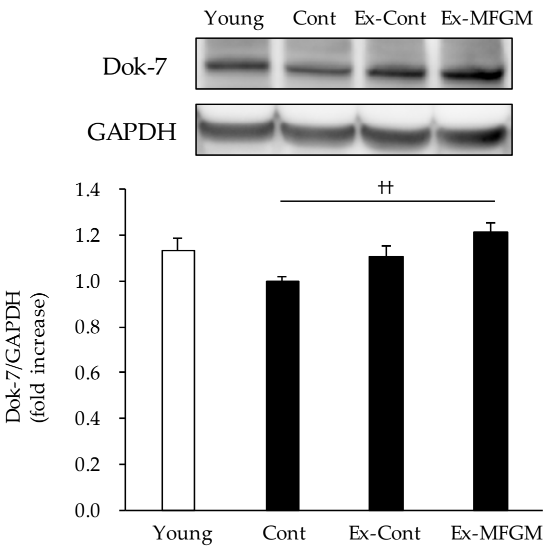

Figure S1. Effect of MFGM with exercise from an old age on the NMJ-related protein Dok-7 in skeletal muscles. Levels of Dok-7 and GAPDH in gastrocnemius muscles were quantified using western blotting at 10 months old (Young) and 24 months old (Cont, Ex-Cont, Ex-MFGM). Values are means ± SE (n = 7-9 in each groups). †† p < 0.01 vs. Cont group by Tukey's test. MFGM, milk fat globule membrane.

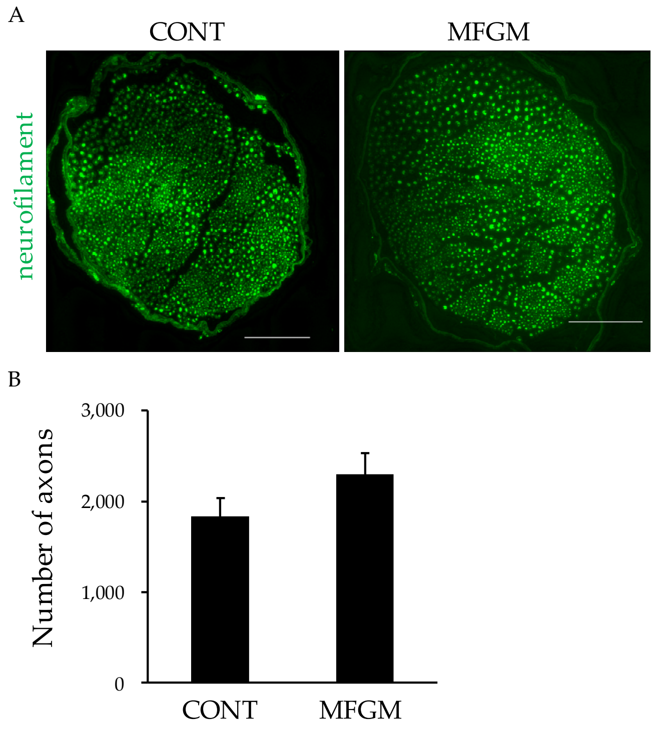

Figure S2. Effect of MFGM from a young age on axon number. Representative images of tibial nerve immunostained with neurofilament were shown (A). The number of axons was counted (B). Values are means ± SE (n = 4-5 in each groups). [Scale bar: 100 μm] MFGM, milk fat globule membrane.

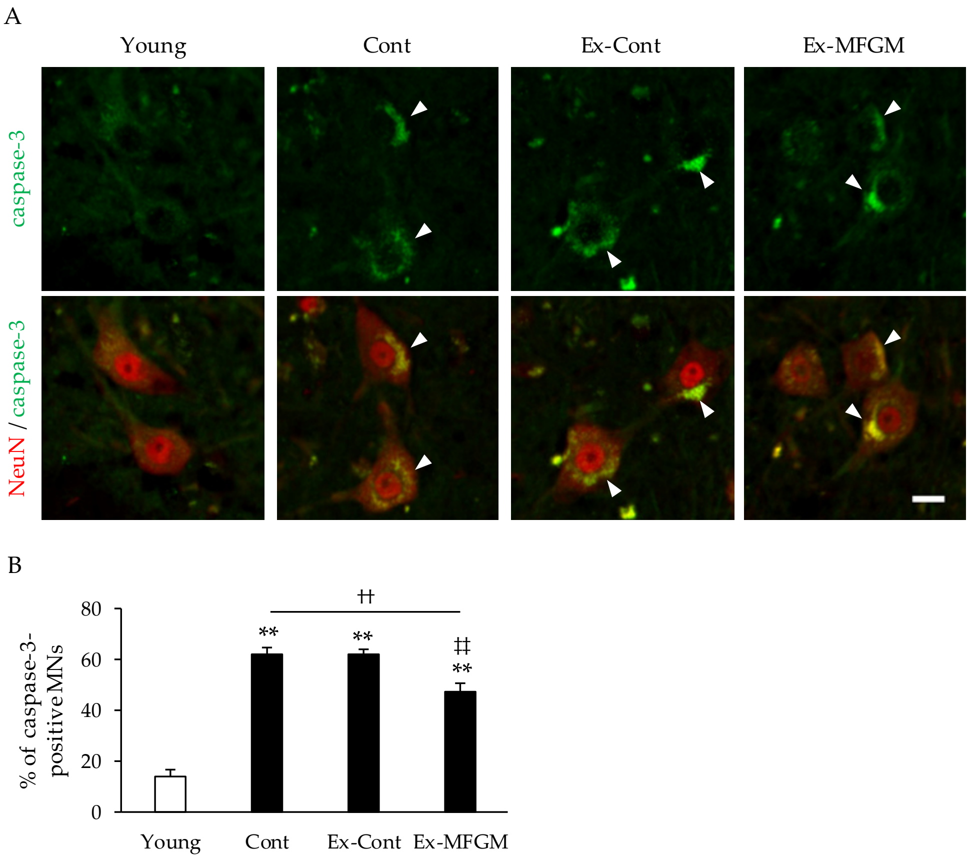

Figure S3. Effect of MFGM with exercise from an old age on caspase-3 expression in somata of motor neurons. Representative images of the spinal cord ventral horn of each group immunostained with cleaved caspase-3 (green) and NeuN (red) were shown at 10 months old (Young) and 24 months old (Cont, Ex-Cont, and Ex-MFGM) (A). The percentage of caspase-3 positive MNs (white arrowheads) was counted (B). Values are means ± SE (n = 6-8 in each groups). ** $p < 0.01$ vs. Young group, †† $p < 0.01$ vs. Cont group, ‡‡ $p < 0.01$ vs. Ex-Cont by Tukey's test. [Scale bar: 20 μm] MFGM, milk fat globule membrane; MN, motor neuron.
